# Does Intravitreal Dopamine Agonist and Antagonist Administration Have Effects on the Brain? An Experimental Study in Rats

**DOI:** 10.5152/eurasianjmed.2022.21288

**Published:** 2022-02-01

**Authors:** Mehmet Nuri Kocak, Orhan Ates, Ahmet Hacımuftuoglu, Irem Ates, Erdal Tekin, Onur Ceylan, Ufuk Okkay

**Affiliations:** 1 Department of Neurology, Atatürk University School of Medicine, Erzurum, Turkey; 2 Department of Ophthalmology, Atatürk University School of Medicine, Erzurum, Turkey; 3 Department of Medical Pharmacology, Atatürk University School of Medicine, Erzurum, Turkey; 4 Department of Anaesthesiology and Reanimation, Atatürk University School of Medicine, Erzurum, Turkey; 5 Department of Emergency Medicine, Atatürk University School of Medicine, Erzurum, Turkey; 6 Department of Medical Pathology, Atatürk University School of Medicine, Erzurum, Turkey

**Keywords:** Basal ganglia, dopamine agonists, dopamine antagonists, intravitreal

## Abstract

**Objective:** There might be dopaminergic connections between the retina and the brain. In this context, the study was aimed to investigate the possible interaction between the retina and basal ganglia through the dopaminergic system.

**Materials and Methods:** In total, 32 healthy rats were randomized into 4 groups: healthy, Sham, dopamine antagonist injected group (risperidone, 0.04 mg/kg intravitreally), and dopamine agonist injected group (apomorphine, 0.4 mg/kg intravitreally). The locomotor activity and Morris water maze tests were applied to all rats twice, before the injection and 28 days after, to detect changes in movement, memory, and attention. Histopathologically, the basal ganglia and hippocampus regions were removed and examined.

**Results:** In the locomotor activity test, a statistical significance was found between the first and last measurement values of the apomorphine group and a decrease in activities and an increase in resting times (*P* < .05). In the Morris water maze test, a statistical significance was detected between the first and last tests of the control group and the apomorphine groups and showed significantly shorter learning times (*P* < .05). Histological analyses of the substantia nigra and hippocampus were noteworthy in that the number of damaged neurons in the risperidone group was considerably higher than the other groups. The number of damaged neurons in the apomorphine group was significantly lower than in the healthy group.

**Conclusion:** Intravitreal administration of dopamine agonists and antagonists has given rise to alterations in the cerebral dopaminergic system, leading to changes in locomotor activity and memory and histopathological changes.

## Main Points

- It was aimed to find the interaction between the retina and basal ganglia by investigating the dopaminergic changes in the retina that may cause similar changes in the cerebral dopaminergic system.
- Intravitreal administration of dopamine agonists and antagonists to the retina was found to cause locomotor activity, memory and histopathological changes in rats.
- With these data we have obtained, it was determined that cerebral changes are possible through the retinal dopaminergic system.

## Introduction

Basal ganglia play an important role in the control of physical motor movements through dopamine. Dopamine is a neurotransmitter that modulates basal ganglia activity in the initiation and execution of targeted movements and habits. Besides, dopamine, as a neuromodulator, plays a key role in motivation and reward-related learning.^1,2^

Dopaminergic dysfunction can cause many pathological conditions such as Parkinson's disease, schizophrenia, depression, Tourette's syndrome, drug and substance abuse.^2^ In Parkinson's disease, which leads to dopamine production failure, there is a slowdown in the movement due to dopamine reduction, tremor, a decrease in behavioral responses, and movement disorders at the extremity ends. As a result of the activation of dopamine receptors, reward behavior in the brain is strengthened. Many experimental studies have suggested that Parkinson's patients have motion perception disorders due to changes in the retina.^3-5^

The retina originates from the neuroectoderm, like the brain, in the early embryogenic stage of neural formation. Therefore, the retina is considered as an embryonic extension of the brain. Dopaminergic activity in the retina plays a role in the development, growth, and cell death of the retina.^6^ Studies have shown that changes in the retina reflect brain functions and structural integrity and can be used as an indicator of progressive degeneration in the cerebral structure.^7,8^ Taking these approaches into consideration, there might be some interconnections and interactions between retina and brain functions, and therefore, structural changes that may occur in the basal ganglia due to retinal dopaminergic stimulation should be investigated in order to detect reciprocal transport.

Our study postulated that dopaminergic functional or structural changes in the retina may result in similar changes in the cerebral dopaminergic system. We thought that there may be a connection between the dopaminergic system in the retina and the basal ganglia and that the determination of this pathway would actually open new approaches in the treatment of many diseases, such as Parkinson’s. This study was aimed to investigate the interaction between dopaminergic system in the retina and dopaminergic system in the basal ganglia, based on studies investigating the relationship between retinal and neurodegenerative diseases in the literature.

## Materials and Methods

### Ethics Committee Approval

Ethics committee approval was received for this study from Atatürk University Medical Experimental Application and Research Center (Approval date and number: June 27, 2019/7-122).

### Animal Selection

In this study, 32 healthy Wistar male albino rats weighing 180-220 g were used. Rats were obtained from University Medical Experimental Application and Research Center. All rats were housed under equivalent environmental conditions during the entire study and fed ad libitum with the same food. Throughout the life of the rats, a 12-hour light/dark (day–night) cycle was provided. All applications were done in the morning to prevent the rats from affecting the circadian cycle.

### Study Groups

In total, 32 rats are randomized and divided into 4 groups.

Group 1: healthy group, not subjected to any application.

Group 2: control group (Sham), composed of rats with intravitreal saline injections.

Group 3: risperidone group, consists of the rats which were injected with risperidone (a dopamine antagonist) by intravitreal injection into the left eyes of the rats.

Group 4: apomorphine group, consists of rats which were injected with apomorphine (a dopamine agonist) by intravitreal injection into the left eyes of the rats.

### Experiment Protocol

In our study, locomotor activity and Morris water maze tests were used to detect changes in movement, memory, and attention in rats twice, before the injection protocol and 28 days after injections. These tests were applied to all study groups. After the study, all rats were sacrificed, and brain tissues were removed, and basal ganglia and hippocampus regions were examined histopathologically.

### Locomotor Activity Test

The locomotor activity test was carried out in a square plexiglas cage with infrared light sources on each edge. When the experimental animal makes a movement in the cage, it interrupts the communication between the mutual infrared sensors and defines the type of motion that this subject performs and it is recorded by a recorder connected to the device. With the help of this system, the stereotypical, ambulatory, vertical and horizontal activities, rest times, and total distance traveled are recorded.

The stereotypical movements record the movements of the animal such as itching, sniffing, and gnawing. Horizontal motion is the movements that the experimental animal performs in its place without making displacement and erection movements. Vertical movement is a movement of perpendicular motion and is perceived with the help of vertical sensors on the cage. The ambulatory movement is any movement of rats, other than standing inside the cage. As all activities were evaluated separately, rest times and total distance traveled were also recorded.

### Morris Water Maze Test

In this test, a round swimming tank with a diameter of 1.5 m and a height of 50 cm is filled with water up to a height of about 35 cm. Then, the water is dyed with black pigment paint in order to determine that the black platform cannot be visible to the animals. Due to the pigment dye property, it only paints the water black, while it does not paint the animals in any way. The water temperature is fixed to 25ºC by means of fixed heaters located in the lower part of this swimming tank. The tank is divided into 4 equal parts hypothetically. These sections are named northeast, northwest, southeast, and southwest according to directions. The square-shaped platform, each side 12.5 cm, is placed in the same area during the experiment, in the desired part of the swimming tank, about 1 cm below the water. Cartons of different colors and geometric shapes are placed on 3 corners of the flotation tank. In the fourth corner, the researcher himself takes place every day.

All rats are floated for 4 times a day for 4 days. For each day, the rats are dropped into the water tank from 4 different points and the times they find the platform are recorded. If they cannot find the platform after 90 seconds, the experiment is ended. These rats, which cannot find the platform, are placed on the platform by hand and kept there for about 20 seconds. The platform discovery times of all rats for 4 days are recorded in seconds. On the fifth day of the experiment, the platform in the water pool was removed. The rats are left in the pool, and how much time they spent in the section where the platform was located before was recorded.

### Injection Protocol

In our study, the active ingredient specific to the group was applied intravitreally to the left eyes of the treated rats in a single session. Before the application, sedation was achieved by applying inhaler sevoflurane anesthesia to all rats. While there is no intervention in the healthy group; 10 μL of saline were injected into the control group rats; 0.04 mg/kg of risperidone (Risperdal 1 mg/mL solution, Johnson & Johnson Sanitary Materials Industry and Trade Ltd. Co., Istanbul, Turkey) to the third group; and the fourth group, 0.4 mg/kg of apomorphine (apomorphine GO ampoule, 20 mg/2 mL, Gen İlaç Ve Sağlık Ürünleri San. Ve Tic. Ltd. Sti., Ankara, Turkey). Injection was applied intravitreally with 33 G injectors. All test application protocols were performed twice, before intravitreal injections and 28 days after injections.

Risperidone is a second-generation antipsychotic drug that is a potent inhibitor of serotonin 5-HT2 and dopamine D2 receptors.^9^ The solution form of risperidone was preferred in our study, because the vial forms of Risperdal are slow-release forms and are not suitable for use.

Apomorphine is a very potent, non-selective dopamine agonist that acts on both D1 and D2 receptors. Apomorphine has long been used in the treatment of Parkinson’s disease.^10^ Among the dopamine agonists, apomorphine was chosen because the only preparation with a vial form is apomorphine Go.

### Histopathological Examination

After the study, the rats in the whole group were sacrificed under 50 mg/kg thiopental general anesthesia and the brain tissues were removed. The same preoperative preparation, anesthesia, and surgical technique were used for each group. The brain tissue in both hemispheres of the rats was histopathologically removed, and the basal ganglia and hippocampus regions were examined in a light microscope after hematoxylin–eosin staining. Whether there were changes in cross-sections in the experimental groups and the presence of neural degeneration were investigated.

### Statistical Analysis

The statistical analyses were performed with Statistical Package for the Social Sciences 25.0 (IBM SPSS Corp.; Armonk, NY, USA) program. All variables were presented as mean and standard deviations before statistical analysis. Kolmogorov–Smirnov test was used for normal distribution assessment. If the 2 groups are normally distributed, Student *t*-test is used and Mann–Whitney *U* test is preferred if not normally distributed; one-way analysis of variance test is used if the compared group is 3 or more and the distribution is normal, and Kruskal–Wallis variance test is applied when it is not normally distributed. Statistical significance was taken as *P* < .05 in the whole study.

## Results

**Figure 1.**
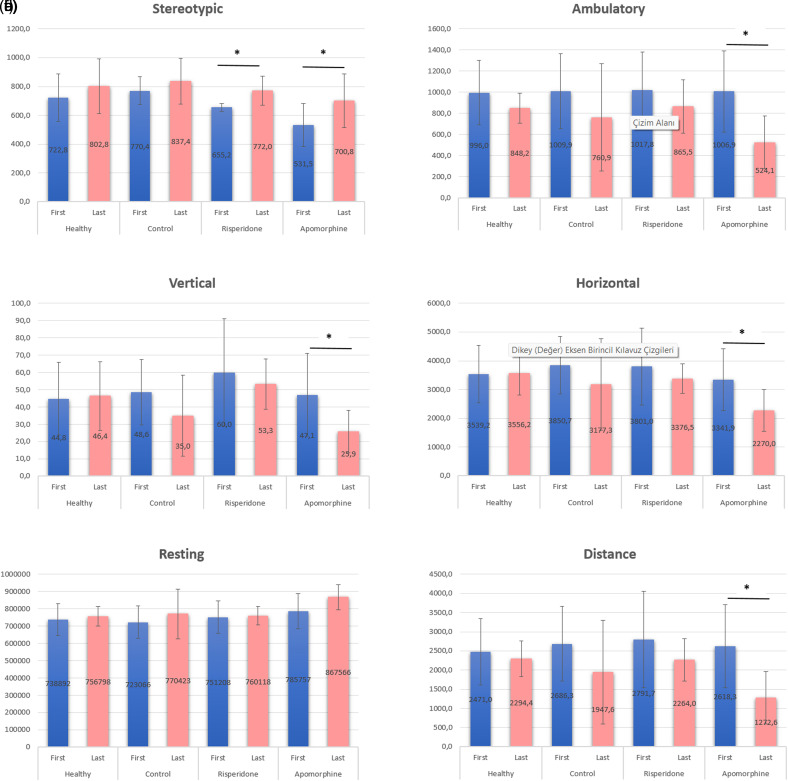
a-f. Comparisons of locomotor activity test results of stereotypic (a), ambulatory (b), vertical (c), horizontal (d), resting times (e), and total distances (f) in the tests. The asterisk above the boxes indicates a statistically significant difference between those 2 groups (*P* < .05).

In the study, stereotypic, ambulatory, vertical, horizontal movements, resting times, and total distances of rats were recorded and compared by conducting the tests twice, before the injection protocol and 28 days after the injection. According to these data, there was no statistically significant difference between the first and last measurements in locomotor activity tests of healthy group and control group rats (*P* > .05). Whereas, in the risperidone group, it was found that there was a decrease in all movements and increased resting time, although only the decrease in stereotypic movements differed statistically significantly (*P* = .021). Also, in the apomorphine group, it was determined that all movements decreased with an increase in resting time, but this difference was found to be statistically significant (*P* < .05). In contrast to the risperidone group, an increase in stereotypic movements was detected in the apomorphine group, and this was statistically significant (*P* = .010) (Figure 1).

**Figure 2.**
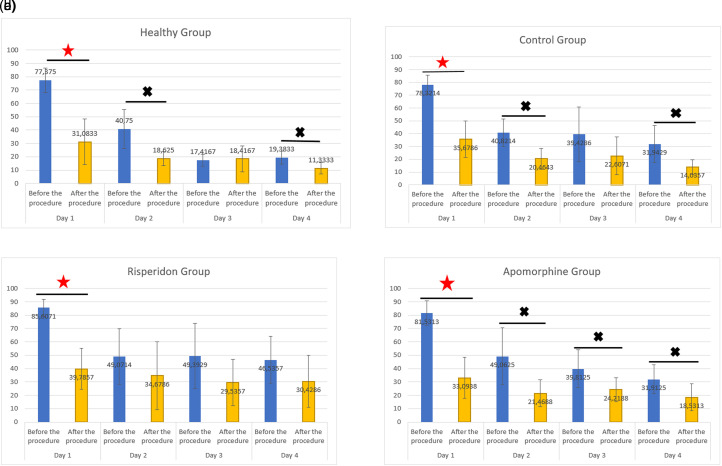
a-d. The comparisons of the first and last day data of healthy (a), control (b), risperidone (c), and apomorphine (d) groups according to Morris water maze tests. The asterisk and cross above the boxes indicate a statistically significant difference between these two groups. (asterisk: *P* < .001; cross: *P* < .05).

In the Morris water maze test, the first 4-day test times of all groups were compared with the 4-day test times performed after the test protocol. According to the data obtained, a significance was found between the first and the last experiment data of the healthy, control, and apomorphine groups, and the rat's time to find the platform in the water shortened significantly (*P* < .05). However, the comparisons of the risperidone group rats on the first day were only significant, while the time to find the platform on the other days was not statistically significant (*P* > .05) (Figure 2).

In another implementation performed in the Morris water maze test, after 4 days of platform discovery times, the platform was removed on the fifth day and the times spent in the platform area were recorded. This test was performed twice, both after 4 days at the start of the study and after 4 days of experimentation after the injection protocol. While the time spent by rats in the platform area was significant in healthy and control groups before and after (*P* < .05), it was found that the risperidone and apomorphine groups were not statistically significant between the groups before and after (*P* > .05). These data demonstrate that dopamine agonist and dopamine antagonist applied groups were found to have negative effects on memory.

**Figure 3.**
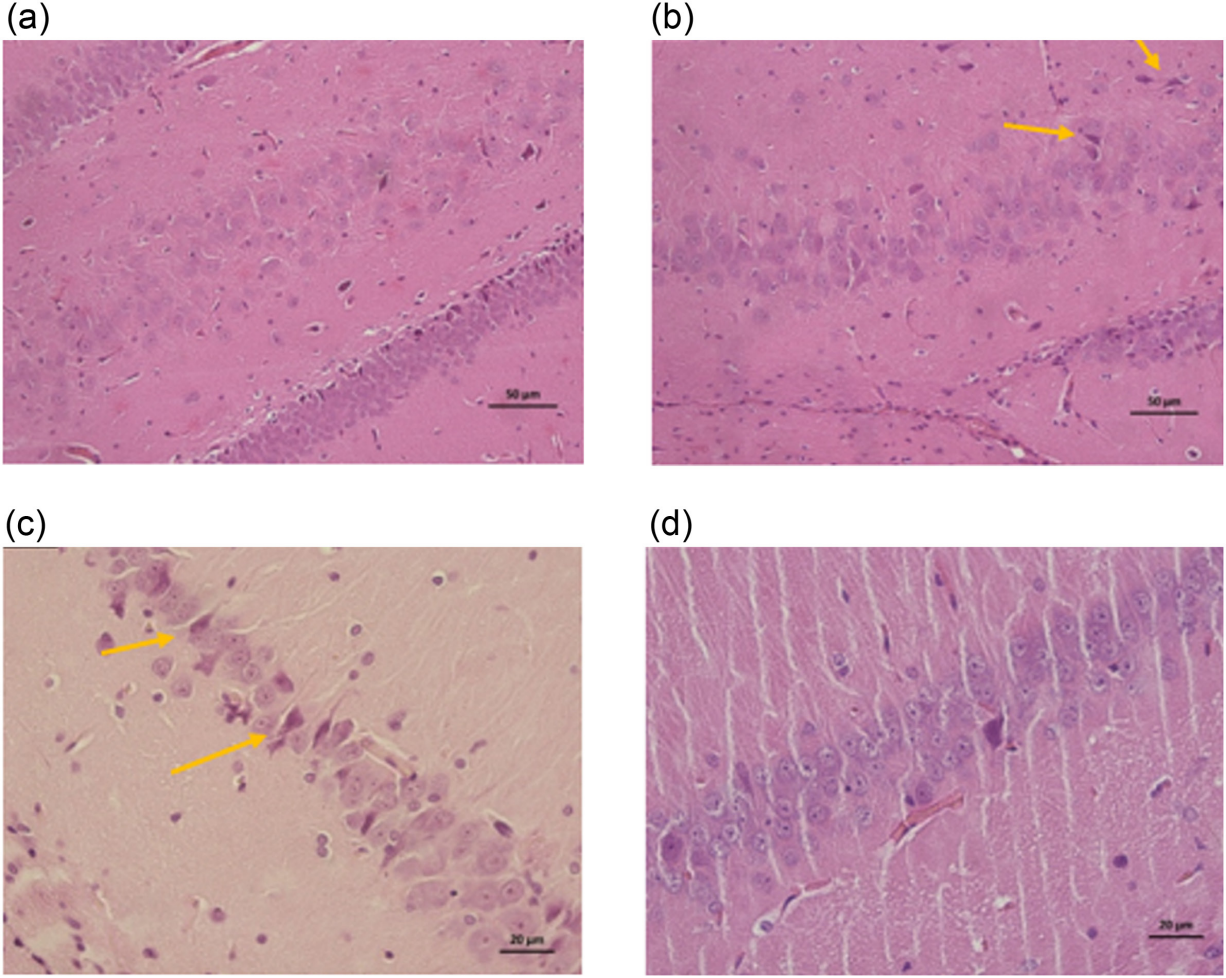
a-d. Histopathologic examination samples of hippocampus of healthy group (a), control group (b), risperidone (c), and apomorphine (d) groups. In healthy group: significantly lower number of degenerated neurons compared to the group receiving risperidone and saline (1-2 pieces). In saline-injected control group: there are very few (2-3) degenerated neurons. In risperidone group: the number of degenerated neurons is much higher than other groups. In apomorphine group: significantly, there are few degenerated neurons.)

**Figure 4.**
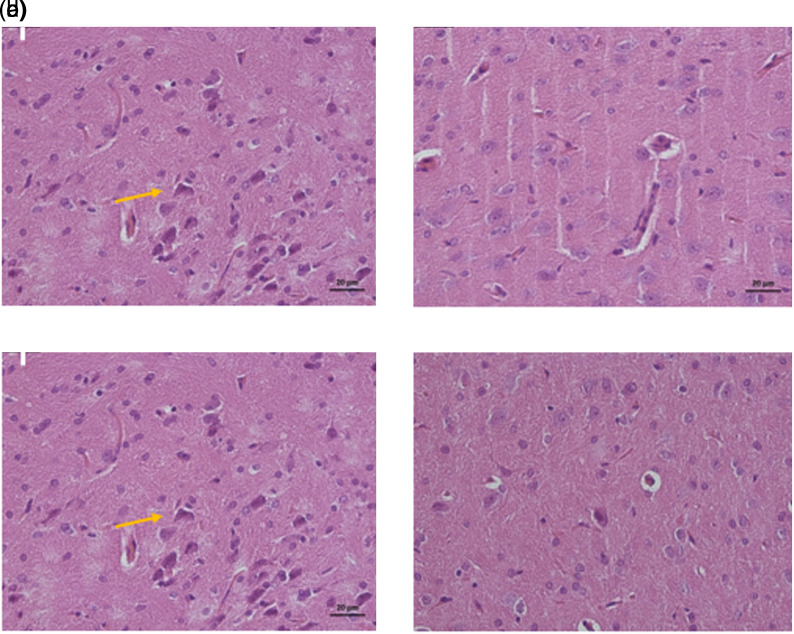
a-d. Histopathologic examination samples of substantia nigra of healthy group (a), control group (b), risperidone (c), and apomorphine (d) groups. In healthy group: significantly lower number of degenerated neurons. In the control group: there are very some degenerated neurons. In risperidone group: the number of degenerated neurons is much higher than other groups. In aqpomorphine group: significantly, there are few degenerated neurons than the other groups.

After the study, the rats were sacrificed and their basal ganglia and hippocampus regions were removed and histopathologically examined (Figure 3 and Figure 4). When we examined sections from substantia nigra and hippocampus, few neurons are found to be damaged or dead in saline-injected control group, whereas, the number of dead or damaged neurons in the group of risperidone has been considerably more numerous than the other groups. In the sections of the apomorphine group, it is observed that the number of dead or damaged neurons is very low, with a significant difference being found with the risperidone group. In fact, the number of dead or damaged neurons in the apomorphine group was found to be quite low, similar to the healthy group. It is noteworthy that the number of dead or damaged neurons in the risperidone group is higher than that of both the saline and apomorphine groups, but, on the other hand, it is lower in the apomorphine group than that of the healthy group. These lead to the conclusion that the apomorphine injected group is protected against neural damage, while the risperidone injected group had increased neural damage and death.

When both the behavioral tests and histopathological data we obtained in our study were gathered, it was concluded that with the application of intravitreal dopamine agonist and antagonist, the retinal dopaminergic system affects the cerebral dopaminergic system, thus leading to behavioral changes, memory changes, and histopathological changes. This result was the first time in the literature to prove that cerebral transport from the retinal dopaminergic system is possible.

## Discussion

Dopamine receptors are commonly found in different parts of the brain and play a role in regulating locomotor movements, working memory, behavior related to reward, desire and pleasure, providing neuronal excitability, and synaptic transmission.^1,4,11^ Dopamine in the retina is released by amacrine and interplexiform cells under the influence of light.^12^ Unlike other cranial nerves, optic nerves are considered as continuation of the brain,^3,5^ and it can be claimed that there have been some connections and interactions between optic nerve and brain. Based on this assumption, the fact that any change in the dopaminergic receptors in the optic nerve-connected retina causes a change in the dopaminergic receptors in the basal ganglion was the starting point of this study.

In this study, the histopathological effects of the experimentally administered intravitreal dopamine agonists and antagonists on the behavior and basal ganglia were investigated and aimed to develop treatments and methods that can be used in schizophrenia, dementia, and Parkinson revealed by degenerations in memory, behavior, and locomotor systems.

All the study outcomes that we gathered, the behavioral tests and histopathological examination results of dopamine agonists and antagonists applied in the retina intravitreally, indicate their transportation and capability of making changes in behavior in rats in basal ganglia, caused by degeneration of neurons or neuroprotection consequences. These results showed us that there is a connection between the retina and the basal ganglia.

The results of locomotor activity and Morris water maze tests were compared before and after intravitreal applications in our study. According to the results of the locomotor activity test, a statistically significance was found between the first and last measurement values of the apomorphine group, as a decrease in activities and an increase in resting times (*P* < .05). The absence of a significant difference in the behavior of the rats in other groups indicates that the intravitreal apomorphine administration via the retinal dopaminergic system was effective on the basal ganglia, thereby causing decreased motion. This situation is the important point of our study, it is concluded that there is neuronal conduction and interaction between the retinal dopaminergic system and basal ganglia dopaminergic system and Parkinsonism symptoms are seen over dopaminergic system in the basal ganglia due to apomorphine application. The absence of locomotor changes in the risperidone group showed compatibility with the result of the literature^13^ that risperidone did not change motor functions in line with the data obtained in the studies. The fact that apomorphine application in our study was obtained as a result of locomotor changes showed that retinal changes can be used for the early diagnosis of diseases such as Parkinson's by interacting with basal ganglia.

In Morris water maze test, which is the second test of our study, the effectiveness of intravitreal risperidone and apomorphine on near memory was investigated and rats were expected to reach the platform in a shorter time. As a result of the study, it was determined that the learning time of the rats was shortened by detecting significant differences between the healthy group, control group, and apomorphine groups between the first application and the last applications (*P* < .05). Although the platform finding time of the risperidone group rats was shortened, it was found to be statistically insignificant. In the tests performed between the fifth day, it was concluded that the risperidone and apomorphine applications caused memory impairments, and intravitreal retinal dopaminergic system resulted in cerebral interaction and consequences.

In the literature, in a study investigating the effects of risperidone on locomotor activity, it was found that risperidone suppresses activity in rats of growth age but causes hyperactivity in adult rats.^14^ This indicates that acute and chronic applications of risperidone may cause different results in rats. In our study, risperidone had no significant effect on locomotor activity.

In a study investigating the effects of apomorphine on locomotor movements, it was determined that apomorphine administered subcutaneously in rats caused hyperactivity.^15^ In our study, although there was an increase in stereotypical movements with intravitreal apomorphine, a decrease was observed in other movements, and this situation showed a statistically significant difference, and a different result was obtained by differentiating from the literature.

In studies investigating the effect of risperidone on memory in the literature, it was found that oral and subcutaneous risperidone impairs the recognition and working memory of the rats.^16,17^ It is seen that the intravitreal risperidone application that we performed with the results of these studies is in parallel with the result that it negatively affects learning and memory.

In a study investigating the effects of apomorphine on memory and learning, it was found that low-dose apomorphine had negative effects on learning and memory, but medium and high dose had no negative effects on memory.^18^ In our study, although we gave 0.4 mg/kg apomorphine at a dose similar to the 0.5 mg/kg dose stated in this study, no negative memory change due to apomorphine was detected. In another study, memory problems were detected in mice receiving 1 mg/kg apomorphine for 15 days.^11^ This led to the conclusion that apomorphine in which we applied a single dose was not given frequently enough to cause sufficient memory problems due to low dose and single session administration.

### Limitations

This study has limitations. Intravitreal injection of dopamine agonists and antagonists can enter or spread into the bloodstream. Absorption rates after intravitreal administration are unknown. However, the doses that we applied are low doses. Therefore, intravitreal administration of low-dose dopamine agonist and antagonist will not be expected to cause similar or the same effects as their systemic administration. Another limitation is that since our study was a preliminary animal experimental study, clinical implications could not be investigated by making a neurodegenerative disease model.

In conclusion, it was obtained for the first time in the literature that the application of dopamine agonists and antagonists on the retinal dopaminergic system caused changes in the basal ganglion dopaminergic system, leading to changes in locomotor activity, memory, and histopathological changes. With these data we obtained, it is expected that cerebral changes are possible through the retinal dopaminergic system, and this will lead to new studies that will enable the development of new alternative treatment methods and applications for the treatment of neurological diseases.
